# Social media exposure, risk perception, preventive behaviors and attitudes during the COVID-19 epidemic in La Paz, Bolivia: A cross sectional study

**DOI:** 10.1371/journal.pone.0245859

**Published:** 2021-01-22

**Authors:** Diana Reyna Zeballos Rivas, Marinalda Lidia Lopez Jaldin, Blanca Nina Canaviri, Luisa Fabiola Portugal Escalante, Angela M. C. Alanes Fernández, Juan Pablo Aguilar Ticona

**Affiliations:** 1 Public Health Department, Higher University of San Andres, La Paz, Bolivia; 2 Post-graduate Program of Collective Health, Federal University of Bahia, Salvador, Brazil; 3 Scientific Society of Medicine Students, Higher University of San Andres, La Paz, Bolivia; 4 Hospital de Clínicas Dr. Manuel Quintela, University of the Republic, Montevideo, Uruguay; Qazvin University of Medical Sciences, ISLAMIC REPUBLIC OF IRAN

## Abstract

Social media has an important role in diffusion of information, during COVID-19 pandemic it could help to promote preventive behaviors, however its role and the pathway is still unclear.

**Objective:**

To investigate the association among social media exposure, risk perception, preventive behaviors, and attitudes toward the COVID-19 epidemic in Bolivia.

**Methods:**

We launched an online survey in La Paz and El Alto, Bolivia, during April and May 2020. The questionnaire examined: Socio-demographic factors, Social media use, Risk Perception, Preventive behaviors, attitudes and the willingness to use a vaccine if it were available in the context of the COVID-19 epidemic. A logistic regression was used to evaluate factors associated with risk perception and a structural equation model (SEM) was performed to explore the pathway of the relationship among social media exposure, risk perception and preventive behaviors and attitudes.

**Results:**

Among 886 participants, the most were young adults, between 18–25 years old (73.4%) and 577 (65.1%) were female. During the the week before the survey 387 (43.7%) reported be exposure to social media Covid-19 information almost always or always. Moreover 304 (34.3%) were categorized as with a high risk perception. The multivariable analyses show that being female (aOR = 1.5, CI 95% 1.1–2.1) and having high exposure to Covid-19 information on social media (aOR = 2.5, CI 95% 1.3–5.3) were associated with a higher risk perception for Covid-19. Furthermore, SEM results indicated that risk perception is associated with the adoption of preventive behaviors and attitudes (β = 0.605, p < 0.001) including the acceptance of a vaccine if one were available (β = 0.388, p < 0.001).

**Conclusion:**

Social media exposure to COVID-19 information influences the adoption of preventive attitudes and behaviors through shaping risk perception. Understanding the role of social media during the pandemic could help policymakers and communicators to develop better communication strategies that enable the population to adopt appropriate attitudes and behaviors.

## Introduction

In December 2019, the first pneumonia cases caused by an unknown agent were identified in Wuhan, China [1]. Later, it was established that the new entity was a novel type of coronavirus, which received the name of severe acute respiratory syndrome coronavirus 2 (SARS-CoV-2) [2]. Within a few months, this disease spread through the five continents turning the epidemic into a pandemic [3]. In Bolivia, the first case was diagnosed and notified in March, with a rising number of new cases since then [4].

Coronaviruses are similar to the influenza virus, due to its contagion and its high transmissibility and they have triggered epidemics, such as SARS-CoV in 2002–2004 [5] and the MERS-CoV in 2015 [2, 6]. The experience of these previous epidemics shows that a modification in behavior to adopt protective measures is required, such as the use of masks, washing hands, and isolation, principally among the affected populations [7]. Furthermore, social media has become a firsthand information channel during epidemics and more so in the pandemic. People can obtain new data about the disease and the current situation to share it with others. Its role in previous epidemics has been studied, demonstrating that social media information can influence people’s own risk perception and behaviors [8, 9]. In this new scenario, the traditional media has reported the progression of the Covid-19 pandemic and social media has assumed an important role in the faster diffusion of information and further and in some cases with fear-mongering [10]. The challenge with such an infodemic we are experiencing is not to get people informed but to get people informed with accurate information that enables them to act properly [11]. Risk perception would motivate individuals to adopt new attitudes and behaviors in order to protect their health [12, 13]. A model explored during the MERS epidemics concluded that risk perception was influenced by social media promoting two self-relevant emotions; fear and anger. At the same time, risk perception was also related to the adoption of protective behaviors such as social distancing and mask use [8]. Facing the new pandemic, the role of social media and risk perception in the implementation of protective measures against the COVID-19 is still unknown.

More than a seventeen million COVID-19 cases have been confirmed worldwide. Facing this health emergency, in Bolivia, as in other countries, a nationwide quarantine has been established on a mandatory basis since March 22th, 2020 (Decrees 4196 and 4199) in addition to mask use and hand washing [4, 14] Accomplishment of the control measures was a challenge and its reception has been heterogeneous among the population. We hypothesize that the acceptance of the preventive measures could be explained by risk perception and social media exposure. We therefore aimed to investigate the factors associated with risk perception to COVID-19. We also explored the association among risk perception with preventive attitudes and behaviors during the first stage of this epidemic in La Paz and El Alto.

## Materials and methods

We conducted a cross sectional study in La Paz and El Alto in La Paz department, in Bolivia. La Paz is the second department with highest incidence of COVID-19, corresponding to 12.35% of the cases reported until April 9, 2020 [15]. The study included participants aged 18 years old or older, with residence in either of the cities mentioned above.

We launched an online survey through social media, available since April 29th to May 9th of 2020. The questionnaire was adapted from Oh et al. study [8] and structured in four sections including 1) sociodemographics and clinical history; 2) social media exposure; 3) risk perception, 4) attitudes and behaviors to prevent COVID-19 including the acceptance of a future vaccine.

To assess risk perception we used a 7-item Likert scale, 1 corresponding to totally disagree and 7 to totally agree, for the following questions: The COVID-19 problem is serious to me; I am worried being affected by the new virus; it is probable that I will be affected by COVID-19; I feel that COVID-19 is dangerous. The mean of the four questions was summarized in low (1–3), mild (4–5) and high risk perception (6–7). We assessed attitudes toward COVID-19 asking about hand washing, alcohol gel use, mask use, social distancing (aligned with the current WHO and Bolivian Health Ministry recommendation) using a 7-item Likert Scale. Preventive behaviors toward COVID-19 included mask use and hand wash frequency. Acceptability of a future vaccine for SARS-CoV-2 was evaluated with two questions using a 7-item Likert Scale, these questions were: Do you agree with vaccines as a preventive measure toward diseases? Would you use a covid-19 vaccine if it were available?. A third question asked about their use of flu vaccines: Were you vaccinated towards the flu? with three possible answers: 1) never in the life; 2) yes, this year; 3) yes, but not this year.

Social media exposure to COVID-19 information in the week before the survey was assessed using a Likert scale of 5 points (1 = never and 5 = always) with the following questions: “How much information have you seen about COVID-19 on Facebook, WhatsApp, Twitter or YouTube?”. Furthermore, we evaluated the emotions (fear and anger) caused by the information with a Likert scale going from 1 never to 7 all the time. Other covariates, such as age, gender, schooling, employment status, health clinical history and having a relative infected by COVID-19 were considered for the analysis.

For statistical analysis, we use descriptive measures to summarize the principal results. The bivariate association was evaluated using Pearson’s χ2 test for categorical variables and Mann-Whitney U test for continuous variables and Likert scales variables that did not have a normal distribution. There were defined as significant p-values < 0.05. A multivariable analysis (models that have two or more outcome or dependent variables) was developed in two-stages. First, we performed a logistic regression to evaluate factors associated with high-risk perception, including social media exposure, clinical history, sociodemographic characteristics. Fear and anger were included, adjusting the logistic regression as intermediate variables linked with social media exposure. In a second-stage, a structural equation model (SEM) with confirmatory factorial analysis was used to describe the direct and indirect relationships in our theoretical model that included social media exposure, feelings experienced, risk perception and preventive behaviors and attitudes including the acceptance of a future vaccine. For the SEM analysis, a confirmatory factorial analysis (CFA) was developed and three latent variables were created related to risk perception; preventive attitudes and behaviors; and vaccine acceptance with the questions that evaluated these domains. On the other hand, social media exposure, fear and anger were included as single variables. The analysis was performed using R Statistical Software version 3.1.6 using the tableone package to table generation [16], Stats package to develop the logistic regression [17], and the Lavaan package for SEM analyses [18].

The study was approved by the Emergency Epidemiological Committee formed to respond to the COVID-19 pandemic at the school of medicine from the Higher University of San Andres and conducted in accordance with the guidelines of The Declaration of Helsinki. Participants read and accepted a consent document online before completing the online survey. All participant data were anonymized. This study was conducted after the written consent of the anonymous volunteer participants was obtained.

## Results

A total of 1100 participants answered the questionnaire; we excluded 214 participants who lived outside the study area. Of the 886 participants included, 673 (76.0%) lived in La Paz and 213 (24.0%) lived in El Alto, the most common group age was 18–25 years old (73.4%), 577 (65.1%) were female, 255 (28.8%) had a formal job. Social media exposure to COVID-19 information in the week before the survey was high, only 6.2% said that they did not receive information about the pandemic on social media (Table 1).

**Table 1:** Characteristics of the participants by risk perception. La Paz–Bolivia. 2020.

Characteristics	Total	Low-Moderate RP	High RP	p value
	n = 886	n = 582	n = 304	
La Paz resident, n (%)	673 (76.0)	434 (74.6)	239 (78.6)	0.209
Female, n (%)	577 (65.1)	354 (60.8)	223 (73.4)	<0.001
Age in years				
< 25	650 (73.4)	437 (75.1)	213 (70.1)	ref
25–45	177 (20.0)	110 (18.9)	67 (22.0)	0.2055
> 45	59 (6.7)	35 (6.0)	24 (7.9)	0.218
Secondary schooling, n (%)	569 (64.2)	376 (64.6)	193 (63.5)	0.798
Formal job, n (%)	255 (28.8)	163 (28.0)	92 (30.3)	0.531
Monthly income, n (%)*				
<1 minimum wage	293 (33.1)	195 (33.5)	98 (32.2)	ref
1–2 minimum wage	248 (28.0)	152 (26.1)	96 (31.6)	0.20
>2 minimum wage	345 (38.9)	235 (40.4)	110 (36.2)	0.67
A relative received medical attention for COVID 19, n (%)	38 (4.3)	21 (3.6)	17 (5.6)	0.227
Exposure to COVID 19 content on social media the week before the survey, n (%)				
Never	55 (6.2)	44 (7.6)	11 (3.6)	ref
Rarely / Sometimes	444 (50.1)	309 (53.1)	135 (44.4)	0.06
Almost always / always	387 (43.7)	229 (39.3)	158 (52.0)	0.002
Feeling experience and information about COVID 19 in social media, median (IIQ)				
Anger	2.0 (1.0–4.0)	2.0 (1.0–4.0)	2.0 (1.0–4.0)	0.155
Fear	3.0 (2.0–5.0)	3.0 (2.0–4.0)	4.0 (3.0–6.0)	<0.001

* 1 minimum wage is equal to a 296 USD.

**Fig 1:**
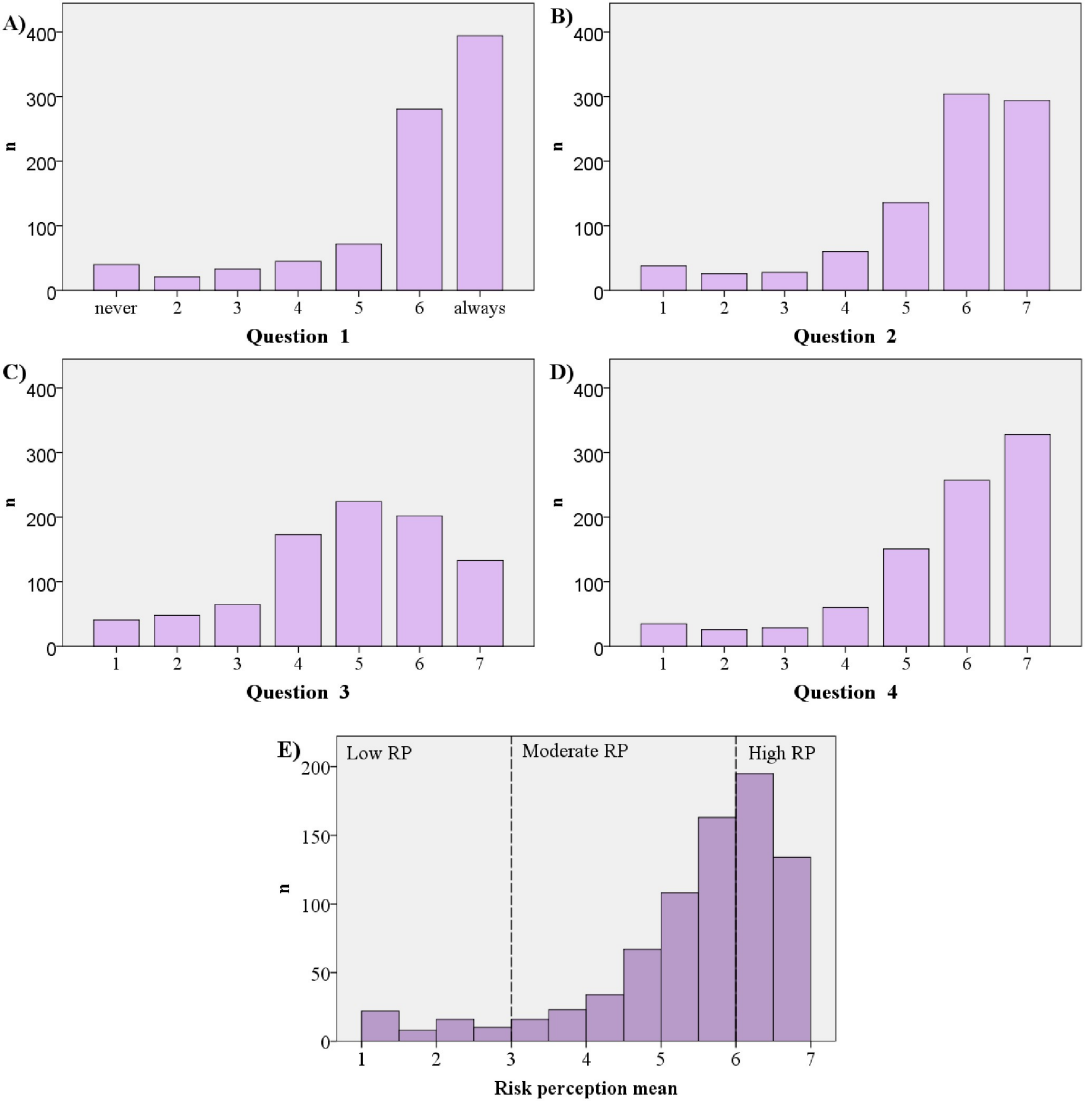
Distribution of the answers related to the participants risk perception. (A) COVID 19 is a serious problem?; (B) I am worried about getting COVID 19?; (C) It is likely that I will get COVID 19?; (D) COVID 19 is dangerous?; and (E) Risk perception mean.

Risk perception to COVID-19 was high in 304 (34.3%) participants and low or moderate in 582 participants (65.7%) (Fig 1 and Table 2). Risk perception was associated with female sex (p<0.001), a high social media exposure (p = 0.002), and fear experience (p<0.001) about COVID 19 information in social media (Table 1). Regarding vaccination, acceptance to a future vaccine for SARS-CoV-2 was high in 481 (54.3%) and moderate in 264 (29.8%) participants. In contrast, this year only 6% of the participants had been vaccinated for influenza and 65.8% had received the vaccine before, but not the current year. Acceptance of a COVID-19 vaccine if one were available was associated with a high risk perception (p<0.001) (Table 3).

**Table 2:** Participants risk perception to COVID-19 in La Paz—Bolivia. 2020.

	Total
	N = 886
Questions related to PR, median (IIQ) *	
COVID 19 is a serious problem	6.0 (6.0–7.0)
I am worried about getting COVID 19	6.0 (5.0–7.0)
It is likely that I will get COVID 19	5.0 (4.0–6.0)
COVID 19 is dangerous	6.0 (5.0–7.0)
Risk perception response average, median (IIQ)	5.8 (5.0–6.5)
Categorized risk PR, median (IIQ)	
Low 1–3	67 (7.6)
Moderate 4–5	515 (58.1)
High 6–7	304 (34.3)

**Table 3:** Vaccination characteristics and acceptance to a SARS-CoV-2 vaccine of the participants in La Paz—Bolivia. 2020.

	Total	Low—Moderate RP	High RP	p value
	n = 886	n = 582	n = 304	
Disposition towards a future vaccine for COVID 19, median (IIQ)	7.0 (5.0–7.0)	6.0 (4.0–7.0)	7.0 (6.0–7.0)	<0.001
Acceptance of a COVID-19 Vaccine if were available.				
Null or Low 1–3	141 (15.9)	110 (18.9)	31 (10.2)	<0.001
Moderate 4–5	264 (29.8)	196 (33.7)	68 (22.4)	<0.001
High 6–7	481 (54.3)	276 (47.4)	205 (67.4)	ref
General acceptance of vaccines, median (IIQ)	6.0 (4.0–7.0)	6.0 (4.0–7.0)	6.0 (5.0–7.0)	<0.001
It received the influenza vaccine, n (%)				
Never	250 (28.2)	179 (30.8)	71 (23.4)	ref
Yes, but not this year	583 (65.8)	372 (63.9)	211 (69.4)	<0.001
Yes, this year	53 (6.0)	31 (5.3)	22 (7.2)	0.03

Participants with positive preventive attitudes (aligned with current recommendations) were more likely to have a high-risk perception (p < 0.001 in all attitudes evaluated). Also, data related to mask use (p <0.001) and high frequency of washing hands (>15 times per day) (p = 0.014) were superior in participants with a high risk perception. The high-risk perception group showed a superior acceptance to vaccination in general (p <0.001) and to a future SARS-CoV-2 vaccine (p <0.001) (Table 4).

**Table 4:** Preventive attitudes and behaviors towards COVID-19 by risk perception in La Paz—Bolivia. 2020.

	Low—Moderate RP	High RP	p value
	n = 582	n = 304	
Prevention attitudes, median (IIQ)*			
Shaking hands	6.0 (4.0–7.0)	7.0 (6.0–7.0)	<0.001
To frequent too crowded places	6.0 (5.0–7.0)	7.0 (6.0–7.0)	<0.001
Use of masks	5.0 (4.0–6.0)	6.0 (5.0–7.0)	<0.001
Washing hands	6.0 (5.0–7.0)	7.0 (6.0–7.0)	<0.001
use of alcohol gel	5.0 (5.0–6.0)	6.0 (5.0–7.0)	<0.001
Quarantine as an effective measure	6.0 (5.0–7.0)	7.0 (6.0–7.0)	<0.001
**Prevention behaviors**			
Use of mask when going when going out of home, median (IIQ)**	5.0 (4.0–5.0)	5.0 (5.0–5.0)	<0.001
Washing hands frequency on the last day, n (%)			
<5	230 (39.5)	108 (35.5)	ref
5–10	275 (47.3)	136 (44.7)	0.741
>10	77 (13.2)	60 (19.7)	0.014

**Fig 2:**
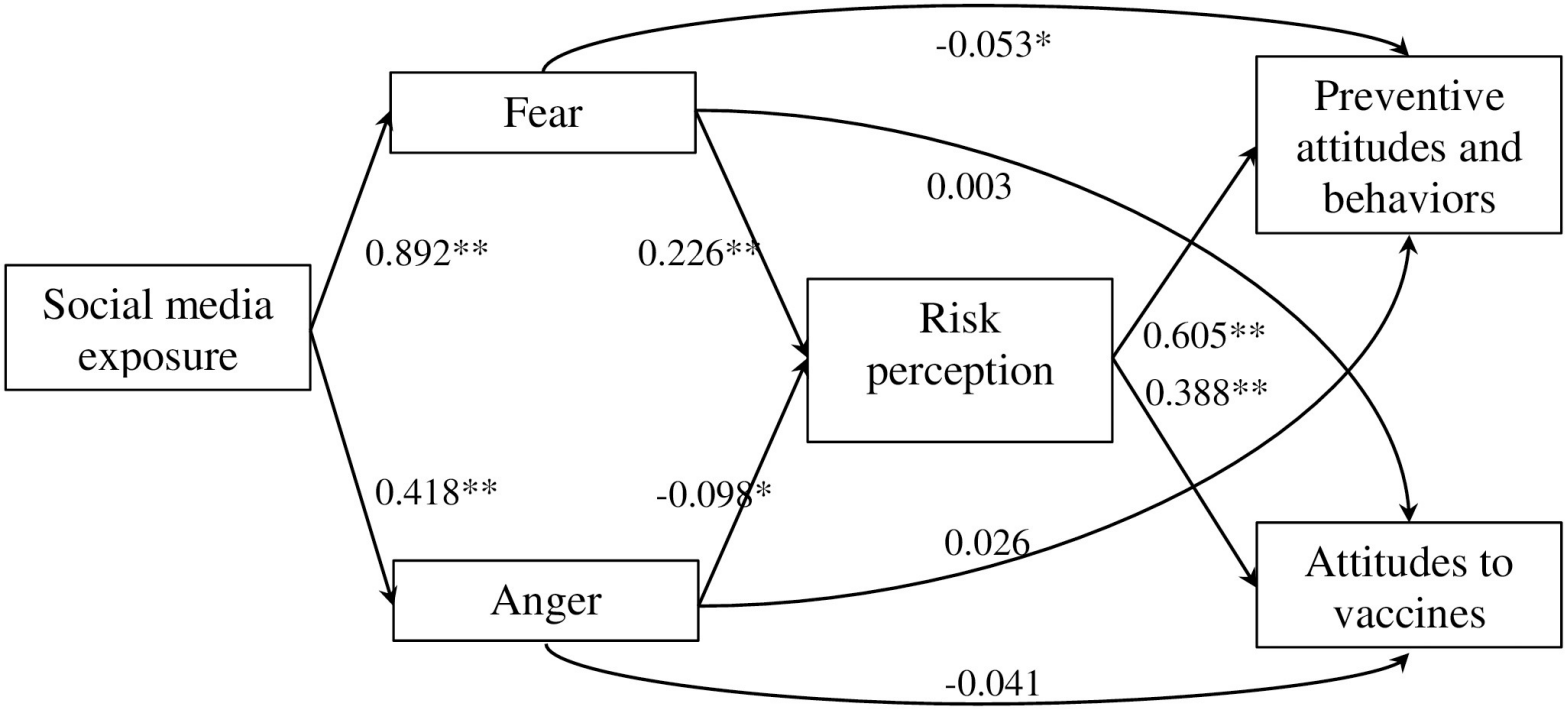
Pathway association. Structural equation model analysis results. * < 0.05; ** <0.001.

In the multivariate analysis, we found that being female increased 50% the chances of having a high risk perception (aOR of 1.5; CI 95% 1.1–2.1). Higher exposure to COVID-19 information on social media was also associated with 2.5 times more chances of having higher risk perception (aOR 2.5; CI95% 1.3–5.3) (Table 5). The Structural equation model had an acceptable fit (RMSEA = 0.030; CFI = 0.989; TLI = 0.986). Factor loadings (β) can be interpreted like a regression coefficient. For each unit increase in a latent variable (risk perception; preventive attitudes and behaviors; and vaccine acceptance) the model predicts a increase with the associated variable if was associated positively or decreases if the association was negative (Fig 2). We could observe that social media exposure is related with fear and anger. Fear was positively related with risk perception (β = 0.226, p < 0.001), while anger was negatively related (β = -0.098, p < 0.005). Risk perception is associated positively with preventive behaviors and attitudes (β = 0.605, p < 0.001), and with the acceptance of a vaccine if one were available (β = 0.388, p < 0.001) (Fig 2, Table 6). Fear was associated with preventive behaviors and attitudes, although this association was negative and weak (β = -0.053, p = <0.001). Other associations in the Structural equation model show the same direction as the binary logistic regression, where social media exposure is indirectly related with risk perception through fear experience (Fig 2). Additional information related to the develop of the three latent (risk perception; preventive attitudes and behaviors; and vaccine acceptance) used in the SEM are summarized in the S1 Table.

**Table 5:** Factors associated with a high risk perception, logistic regression results.

	p value	aOR (IC 95%)
Female	<0.001	1.8 (1.3–2.4)
La Paz resident	0.144	1.3 (0.9–1.8)
Moderate exposure to social media	0.208	1.6 (0.8–3.3)
High exposure to social media	0.010	2.5 (1.3–5.3)

aOR, adjusted Odds Ratio.

**Table 6:** Pathway association of social media exposure, feeling experience, risk perception and preventive attitudes and behaviors. Structural equation model analysis results.

Relationship (effect)			Standard Error	p value	Estimate (β)	CI 95%
Fear	~	Attitudes and behaviors	0.021	<0.001	-0.053	(-0.15– -0.04)
Anger	~	Attitudes and behaviors	0.022	0.409	0.026	(-0.04–0.09)
Risk perception	~	Attitudes and behaviors	0.046	<0.001	0.605	(0.56–0.65)
Fear	~	Vaccine acceptance	0.023	0.943	0.003	(-0.07–0.07)
Anger	~	Vaccine acceptance	0.021	0.244	-0.041	(-0.11–0.03)
Risk perception	~	Vaccine acceptance	0.038	<0.001	0.388	(0.33–0.45)
Fear	~	Risk perception	0.021	<0.001	0.226	(0.16–0.29)
Anger	~	Risk perception	0.020	0.005	-0.098	(-0.17–-0.03)
Social media exposure	~	Fear	0.249	<0.001	0.889	(0.60–1.17)
Social media exposure	~	Anger	0.127	<0.001	0.418	(0.29–0.55)

## Discussion

We found that high exposure to COVID-19 information on social media is associated with higher risk perception, moreover, fear and anger could influence the shaping of this risk perception. Women were 50% more likely to perceive themselves at risk to COVID-19 when compared to men. Higher risk perception is associated with preventive behaviors and attitudes, including a SARS-CoV-2 vaccine acceptance. The adoption of preventive behaviors and control measures has been fundamental to overcoming previous epidemics, but the success of these actions relied on the population response. Our findings corroborate the literature, risk perception is an important factor that influences the population’s willingness to adopt behaviors such as mask use, frequent hand washing and physical distancing [19–21], especially in a scenario surrounded by uncertainties at the beginning of the epidemic in Bolivia.

During the MERS outbreak in 2015, a study determined that social media exposure to the outbreak information influenced risk perception and population behaviors [8, 9]. Furthermore, similar to our results, social media exposure was positively associated with fear and anger [8]. Experiencing fear during COVID-19 pandemic has been previously reported, and also associated with positive preventive behaviors [22–24]. We also observed that fear was positively associated with risk perception, while anger was negatively associated with risk perception. Anger is related to an optimistic position regarding potential risk [25], as consequence people are confident in controlling the risk and tend to minimize their own risk [8, 21, 25]. Even though COVID-19 was an uncertain and uncontrollable for a while, by the time that the first cases were reported in Bolivia, information about successful experiences of other countries was available [26]. We also observed that fear had a stronger influence on risk perception when compared with anger, as has been previously described in literature [8]. Depoux et al. recommend social media as a tool for information, and in China it was used as a disclosure tool for quarantine, giving advice to the population. Also, social media can help reduce social distancing and mental health problems encountered by people forced into quarantine [10, 25].

Females were more likely to perceive themselves at risk, which is consistent with other studies conducted in previous epidemics [8, 20]. A recent study that surveyed people of ten countries on risk perception of COVID-19 found that being male was associated with lower risk perception and the same pattern was observed in many countries [27]. This is interesting because the novel coronavirus tends to have a more severe and deadly presentation in men. It is unclear whether these higher fatality rates derive from biological or behavioral differences, or if it is due to the heterogeneity of the current data [28, 29]. However, male risky behaviors such as ignoring health preventive measures, smoking and not considering symptoms seriously could be contributing to this difference [29].

Acceptance of a vaccine for SARS-CoV-2 if available, 54% of the participants thought they would and 30% thought they might. The structural equation analysis showed that acceptability is associated with risk perception; it means those who perceived higher risk were more likely to accept the vaccine. As described before, risk perception is heterogeneous and varies according to the disease, the context, and participants. A study conducted in Germany explored attitudes toward the Ebola vaccine and the findings showed a low proportion of people who would actually use the vaccine [30]. These results can stem from the fact that the Ebola epicenter stayed in Africa. A study conducted in Poland where no cases of Èbola were registered, 97% of the participants affirmed that Èbola disease was mortal but just 15% would use a vaccine [31]. This scenario would change if they traveled to to a country where this disease were endemic, 92.5% affirmed that in that case they would use the vaccine [31]. People had the information but the mediator to take an action was risk perception. A recent study investigated the willingness to be vaccinated against COVID-19 in France, a country where vaccination was widely rejected. The authors observed that 77.6% of the participants would certainly or probably use the vaccine. The acceptance of a future vaccine was associated with individual risk perception, principally in those groups that are known to be high-risk such as health care workers and the elderly [32]. However, this scenario can change if the number of cases decreases when COVID-19 vaccine becomes available. It is surprising that barely 6% of the participants declared that they had received the influenza vaccine this year and only 65.8% indicated that they had had it sometime in their lives. A study that assessed the relationship between social media use and influenza vaccine uptake found that despite the fact that social media was not a main source of health information, it has a potential role in shaping behaviors to increase influenza vaccination rates [12]. Furthermore, influenza vaccination lessons could be useful when COVID-19 vaccines become available [33], as we could observe, a high percentage of this Bolivian population had been vaccinated against the flu virus before.

Limitations of our study are related to the study design that did not allow us to establish a causal relationship. As we conducted an on-line survey accessibility issues should be addressed, representativity of our population might be limited to those who have access to the internet. In consequence, our sample had a high number of young adults. However, an online survey is a strategy that in the current context gives us the opportunity to do primary research and find a population exposed to social media. The results of this study correspond to the first time period of the COVID-19 in Bolivia; other studies need to be performed to understand the dynamic of attitudes and behaviors during the period of the highest numbers of infections and after the epidemic. We did not evaluate the quality of the content to which the participants were exposed as this could also influence behaviors even negatively [10], this also can be associated with mental health problems [34]. In consequence future studies need to be conducted to understand the influences of the content quality and the association with the factors we addressed. Understanding the determinants of the perception of risk among people is critical to disseminate information on appropriate public health behaviors [35].

In conclusion, our results address an important issue with social media as an information channel and its association with shaping risk perception and behaviors to communicate information to the population. Effective communication with the population by public health agencies and governments is among the most important components of successful pandemic responses. Successful communication can help the public adopt appropriate behaviors to stop the spread of an outbreak. This findings presented can help policymakers and communicators better understand the complex process of emotions and cognition provoked by infectious disease outbreaks and develop better communication strategies.

## Supporting information

S1 Table Latent variables built and components calculated with confirmatory factor analysis. (DOCX)

S1 File Survey instrument. (DOCX)
