# Supplementary material for: Social media exposure, risk perception, preventive behaviors and attitudes during the COVID-19 epidemic in La Paz, Bolivia: A cross sectional study
Source: PLoS One. 2021 Jan 22;16(1):e0245859. doi: 10.1371/journal.pone.0245859 (PMC7822287; doi:10.1371/journal.pone.0245859)
Supplement: S1 Table — (DOCX) [file pone.0245859.s001.docx]

S1 Table. Latent variables built and components calculated with confirmatory factor analysis.

| Latent variables | Components | B | SE | Z | p-value | Coefficient |
| --- | --- | --- | --- | --- | --- | --- |
| Risk perception | COVID 19 is a serious problem? | 1,39 | 0,06 | 23,08 | < 0,001 | 0,89 |
|  | I am worried about getting COVID 19? | 1,29 | 0,05 | 25,05 | < 0,001 | 0,85 |
|  | It is likely that I will get COVID 19? | 0,92 | 0,05 | 16,78 | < 0,001 | 0,59 |
|  | COVID 19 is dangerous? | 1,28 | 0,05 | 23,82 | < 0,001 | 0,84 |
| Attitudes and behaviors | Use of mask when going when going out of home | 0,17 | 0,03 | 6,56 | < 0,001 | 0,22 |
|  | Washing hands frequency on the last day | 0,09 | 0,03 | 2,77 | < 0,001 | 0,11 |
|  | Avoid shaking hands | 1,16 | 0,06 | 19,08 | < 0,001 | 0,79 |
|  | Avoid to frequent too crowded places | 1,19 | 0,06 | 20,76 | < 0,001 | 0,86 |
|  | Use of masks | 0,97 | 0,04 | 21,53 | < 0,001 | 0,75 |
|  | Washing hands | 1,10 | 0,04 | 24,48 | < 0,001 | 0,87 |
|  | Use of alcohol gel | 1,03 | 0,04 | 24,60 | < 0,001 | 0,83 |
|  | Quarantine as an effective measure | 1,10 | 0,06 | 18,59 | < 0,001 | 0,79 |
| Vaccine acceptance | General acceptance of vaccines | 1,48 | 0,09 | 15,88 | < 0,001 | 0,79 |
|  | Disposition towards a future vaccine for COVID 19 | 1,75 | 0,12 | 14,85 | < 0,001 | 0,92 |
