# Supplementary material for: Social media exposure, risk perception, preventive behaviors and attitudes during the COVID-19 epidemic in La Paz, Bolivia: A cross sectional study
Source: PLoS One. 2021 Jan 22;16(1):e0245859. doi: 10.1371/journal.pone.0245859 (PMC7822287; doi:10.1371/journal.pone.0245859)
Supplement: S1 File — (DOCX) [file pone.0245859.s002.docx]

S1 File. Survey instrument.

This survey was designed by the research team. Data collection occurred between April 29th to May 9th via an online survey with a sample of adults in the cities of La Paz and El Alto, Bolivia. Please cite the associated article when referencing this survey.

| ID | sections | Item | Response Scale |
| --- | --- | --- | --- |
| A1 | Sociodemographics and clinical history | What is your age? | ____________ years old |
| A2 | Sociodemographics and clinical history | What is your nationality? | ____________ |
| A3 | Sociodemographics and clinical history | What city do you currently live in? | ____________ |
| A4 | Sociodemographics and clinical history | Which is your sex? | 1 – Male  2 – Female  X BLANK |
| A5 | Sociodemographics and clinical history | What is the highest level of education that you have complete? | 1 – Less than a Secondary school degree  2 – Secondary school degree  3 – Some college or technical school  4 – College degree or more  X BLANK |
| A6 | Sociodemographics and clinical history | Which is your employment status? | 1 – Formal job  2 – Informal job  3 – Unemployed  X BLANK |
| A7 | Sociodemographics and clinical history | Monthly income | ____________bolivianos |
| A8 | Sociodemographics and clinical history | Do you have a relative that received medical attention for COVID-19? | 1 – Yes  2 – No  3 – If yes. Who? ____________  X BLANK |
| A9 | Sociodemographics and clinical history | How much have you seen information about COVID-19 on social media, such as on Facebook, WhatsApp, Twitter or YouTube? | 1 – Never  2 – Rarely/Almost never  3 – Sometimes  4 – Almost always  5 – Always |
| RP1 | Risk perception | The COVID-19 problem is serious to me | 1 – Strongly disagree  2 – Disagree  3 – Somewhat disagree  4 – Neither agree or disagree  5 – Somewhat agree  6 – Agree  7 – Strongly agree |
| RP2 | Risk perception | I am worried being affected by the new virus | 1 – Strongly disagree  2 – Disagree  3 – Somewhat disagree  4 – Neither agree or disagree  5 – Somewhat agree  6 – Agree  7 – Strongly agree |
| RP3 | Risk perception | It is probable that I will be affected by COVID-19 | 1 – Strongly disagree  2 – Disagree  3 – Somewhat disagree  4 – Neither agree or disagree  5 – Somewhat agree  6 – Agree  7 – Strongly agree |
| RP4 | Risk perception | I feel that COVID-19 is dangerous. | 1 – Strongly disagree  2 – Disagree  3 – Somewhat disagree  4 – Neither agree or disagree  5 – Somewhat agree  6 – Agree  7 – Strongly agree |
| E1 | Emotions | I am fearful of COVID-19 | 1 – Not at all  2 – Low  3 – Slightly  4 – Neutral  5 – Moderately  6 – Very  7 – Extremely |
| E2 | Emotions | I am frightened by COVID-19 | 1 – Not at all  2 – Low  3 – Slightly  4 – Neutral  5 – Moderately  6 – Very  7 – Extremely |
|  | Attitudes and behaviors | Do you agree with the following statements to prevent COVID-19? | Response Scale |
| AB1 | Attitudes and behaviors | Avoid shaking hands | 1 – Strongly disagree  2 – Disagree  3 – Somewhat disagree  4 – Neither agree or disagree  5 – Somewhat agree  6 – Agree  7 – Strongly agree |
| AB2 | Attitudes and behaviors | Avoid to frequent too crowded places | 1 – Strongly disagree  2 – Disagree  3 – Somewhat disagree  4 – Neither agree or disagree  5 – Somewhat agree  6 – Agree  7 – Strongly agree |
| AB3 | Attitudes and behaviors | Use of masks | 1 – Strongly disagree  2 – Disagree  3 – Somewhat disagree  4 – Neither agree or disagree  5 – Somewhat agree  6 – Agree  7 – Strongly agree |
| AB4 | Attitudes and behaviors | Washing hands | 1 – Strongly disagree  2 – Disagree  3 – Somewhat disagree  4 – Neither agree or disagree  5 – Somewhat agree  6 – Agree  7 – Strongly agree |
| AB5 | Attitudes and behaviors | Use of alcohol gel | 1 – Strongly disagree  2 – Disagree  3 – Somewhat disagree  4 – Neither agree or disagree  5 – Somewhat agree  6 – Agree  7 – Strongly agree |
| AB6 | Attitudes and behaviors | Quarantine as an effective measure | 1 – Strongly disagree  2 – Disagree  3 – Somewhat disagree  4 – Neither agree or disagree  5 – Somewhat agree  6 – Agree  7 – Strongly agree |
| AB7 | Attitudes and behaviors | How often do you use mask when going out of home? | 1 – Never  2 – Rarely  3 – Occasionally  4 – Sometimes  5 – Frequently  6 – Usually  7 – Every time |
| AB8 | Attitudes and behaviors | Washing hands frequency on the last day | N°____________ |
| V1 | Attitudes and behaviors | Do you agree with vaccines as a preventive measure toward diseases? | 1 – Strongly disagree  2 – Disagree  3 – Somewhat disagree  4 – Neither agree or disagree  5 – Somewhat agree  6 – Agree  7 – Strongly agree |
| V2 | Attitudes and behaviors | If a COVID-19 vaccine is proven safe and effective and is available to me, I will take it? | 1 – Strongly disagree  2 – Disagree  3 – Somewhat disagree  4 – Neither agree or disagree  5 – Somewhat agree  6 – Agree  7 – Strongly agree |
| V3 | Attitudes and behaviors | Were you vaccinated towards the flu? | 1 – Never in the life;  2 – Yes, this year;  3 – Yes, but not this year |
